# Evaluation of force feedback in walking using joint torques as “naturalistic” stimuli

**DOI:** 10.1152/jn.00120.2021

**Published:** 2021-06-09

**Authors:** Sasha N. Zill, Chris J. Dallmann, Nicholas S. Szczecinski, Ansgar Büschges, Josef Schmitz

**Affiliations:** ^1^Department of Biomedical Sciences, Joan C. Edwards School of Medicine, Marshall University, Huntington, West Virginia; ^2^Department of Physiology and Biophysics, University of Washington, Seattle, Washington; ^3^Department of Mechanical and Aerospace Engineering, West Virginia University, Morgantown, West Virginia; ^4^Department of Animal Physiology, Institute of Zoology, Biocenter, University of Cologne, Cologne, Germany; ^5^Department of Biological Cybernetics, Bielefeld University, Bielefeld, Germany

**Keywords:** load, posture, sensory encoding, walking

## Abstract

Control of adaptive walking requires the integration of sensory signals of muscle force and load. We have studied how mechanoreceptors (tibial campaniform sensilla) encode “naturalistic” stimuli derived from joint torques of stick insects walking on a horizontal substrate. Previous studies showed that forces applied to the legs using the mean torque profiles of a proximal joint were highly effective in eliciting motor activities. However, substantial variations in torque direction and magnitude occurred at the more distal femorotibial joint, which can generate braking or propulsive forces and provide lateral stability. To determine how these forces are encoded, we used torque waveforms of individual steps that had maximum values in stance in the directions of flexion or extension. Analysis of kinematic data showed that the torques in different directions tended to occur in different ranges of joint angles. Variations within stance were not accompanied by comparable changes in joint angle but often reflected vertical ground reaction forces and leg support of body load. Application of torque waveforms elicited sensory discharges with variations in firing frequency similar to those seen in freely walking insects. All sensilla directionally encoded the dynamics of force increases and showed hysteresis to transient force decreases. Smaller receptors exhibited more tonic firing. Our findings suggest that dynamic sensitivity in force feedback can modulate ongoing muscle activities to stabilize distal joints when large forces are generated at proximal joints. Furthermore, use of “naturalistic” stimuli can reproduce characteristics seen in freely moving animals that are absent in conventional restrained preparations.

**NEW & NOTEWORTHY** Sensory encoding of forces during walking by campaniform sensilla was characterized in stick insects using waveforms of joint torques calculated by inverse dynamics as mechanical stimuli. Tests using the mean joint torque and torques of individual steps showed the system is highly sensitive to force dynamics (dF/dt). Use of “naturalistic” stimuli can reproduce characteristics of sensory discharges seen in freely walking insects, such as load transfer among legs.

## Graphical abstract

**Figure:**
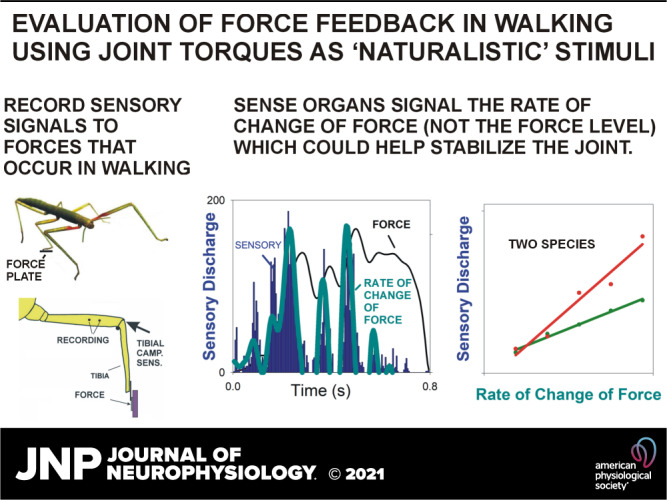

## INTRODUCTION

Regulation of force is considered an integral component in the control of adaptive leg movements in walking (1–4). Muscle forces and loads are monitored mainly by sense organs in the legs and feet (5, 6). However, there are disparate views on the specific parameters of forces that are signaled by these receptors. In vertebrates, for example, early experiments established that the discharges of Golgi tendon organs, which monitor forces through strains at muscle insertions, could encode force magnitude, providing information necessary in the detection and regulation of body load (7–9). However, tendon organs have strong dynamic sensitivities that could compromise linear encoding of force magnitude in walking: receptor discharges were, instead, considered to function mainly to provide signals on variations in forces, and the detection of “static muscle force might require the combined processing of discharges from other types of receptors” (10; see also Ref. 11). Subsequent studies have proposed that information about force magnitude is derived from sensory receptors as a population that converges in the central nervous system, although the specific mechanisms have not been elucidated.

We have studied how forces are detected and controlled in posture and locomotion in insects. Campaniform sensilla are mechanoreceptors that encode forces as strains in the exoskeleton (3, 12–14). Many groups of receptors can be activated by imposed forces but, like Golgi tendon organs, campaniform sensilla specifically encode muscle forces that act against external loads (15, 16). The receptor neurons can not only signal the magnitude of forces under defined experimental conditions but also show strong dynamic sensitivities (17).

To assess the effects of force receptors in walking, a previous study examined motor responses to forces imposed upon the legs using “naturalistic” stimuli of joint torques (18). The torque profiles were calculated by inverse dynamics in experiments that characterized ground reaction forces and leg movements of freely walking stick insects (19). Use of joint torques as stimuli elicited vigorous activation of groups of synergist leg muscles and reduced adaptation that occurred to forces imposed as ramp and hold waveforms. Motor activities could also be elicited by transient perturbations introduced into the waveforms.

However, those experiments only characterize motor activities during application of mean torques at one proximal leg joint (CTr, coxo-trochanteral joint) and did not extensively characterize the effects of other joint torques on sensory discharges. The present study was undertaken to examine sensory encoding of joint torques at the more distal femorotibial (FT) joint. The sensory receptors at the FT joint (tibial campaniform sensilla) are limited in number and can be readily characterized by extracellular recording. The mean torque at the FT joint in walking on a horizontal substrate was quite small in comparison with the proximal articulations (19). However, the torque values were quite variable and could show changes in sign (flexor vs. extensor). The present study sought to characterize sensory encoding of this variability by applying torque waveforms of single steps that were selected based on their deviation from the mean values. The goal of this study was to use variations in the system as “natural” perturbations to examine the properties of sense organs in signaling force amplitude and rate. Although previous studies used sudden changes in waveforms, these stimuli were inherently artificial. Perturbations can also act as “novel” stimuli and elicit components of compensatory reactions (20) that do not reflect the way the system encodes and reacts to normal variations.

In addition, campaniform sensilla of insects occur in groups, and receptors within a cluster show a range fractionation and diverse sensitivities (21, 22). In the present study, analysis of sensory responses included sensilla with smaller extracellular potentials. Those receptors show more tonic activities but are rarely characterized. This approach has confirmed that the system is particularly sensitive to force dynamics during walking but can reflect force magnitude as a population, although all sensilla show hysteresis (23). Evaluation of data on joint angles and ground reaction forces during these steps supports the hypothesis that the dynamic sensitivities in force signals can aid in stabilizing the femorotibial joint to produce smooth movements in walking, whereas larger forces are generated in support and propulsion at the more proximal leg joints (24).

## METHODS

**Figure 1.**
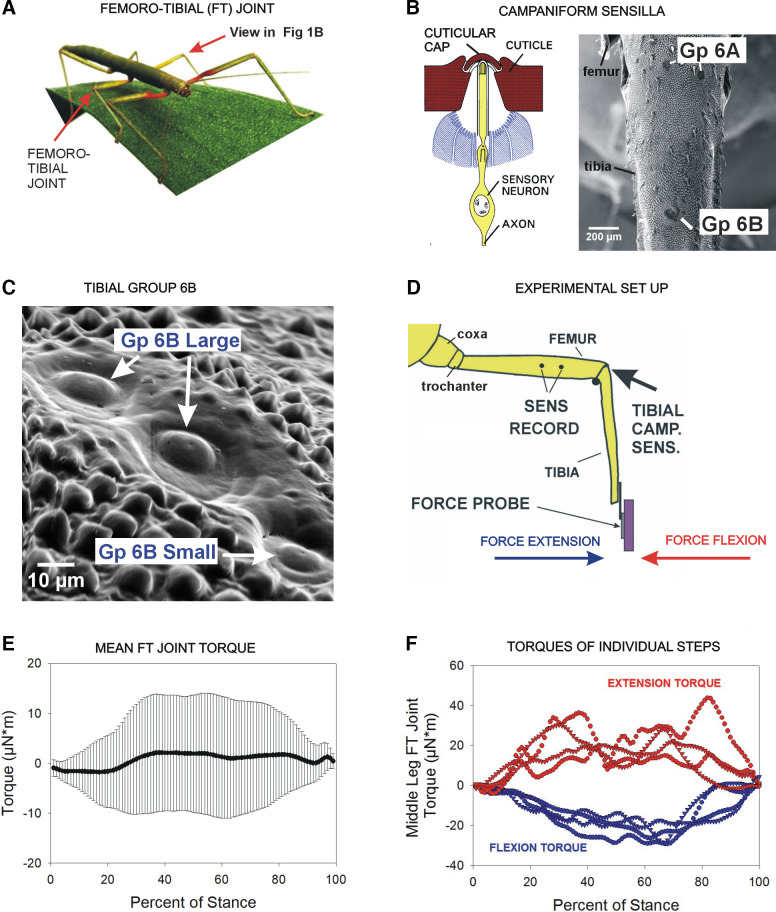
Preparation and torque waveforms. *A*: sensory encoding of torques of the femorotibial (FT) joint was studied in the middle legs of stick insects (red arrows). *B*: *left*—diagram of a campaniform sensillum. Forces are encoded via cuticular strains transmitted to the cuticular cap. *Right*—scanning electron micrograph (SEM) of tibia (see *A* for orientation). Tibial campaniform sensilla consist of two spatially separated subgroups (6A and 6B). *C*: high-power SEM of group 6B that contains sensilla with large and smaller caps. *D*: preparation—activities of the tibial campaniform sensilla were recorded extracellularly in the femur while forces were applied to the tibia using profiles of joint torques (movement resisted). *E*: mean joint torques—mean torque at the FT joint during the stance phase of walking was calculated by inverse dynamics (data from Ref. 19). The values varied considerably (as indicated by the standard deviations). *F*: torques of individual steps—torques that had the maximum averaged values in the directions of flexion and extension were selected, and these profiles were applied as stimuli to characterize sensory encoding in the present study. Gp, group; Tibial camp. sens., tibial campaniform sensilla.

Experiments were performed on the middle legs of adult, female Indian stick insects (*Carausius morosus*, *N* = 15) obtained from colonies at the University of Cologne (Fig. 1*A*). Activities of tibial campaniform sensilla (Fig. 1, *B* and *C*) were also recorded from adult, female Annam stick insects (*Medauroidea extradentata*, *N* = 15) obtained from a commercial supplier (Backwater Reptiles). *Medauroidea extradentata* (syn. Cuniculina impigra) are a species of stick insects similar to *Carausius* but are larger in body size and mass (25).

Data were extensively analyzed from preparations (*N* = 4, *Carausius*; *N* = 4, *Medauroidea*) that showed stability throughout the series of tests, ablations, and retests (monitored by changes in amplitude of extracellular action potentials) and similar stiffness of the cuticle (indicated by the displacement needed to generate resistive forces). In *Carausius*, these recordings permitted consistent detection of activities of sensilla with small extracellular potentials over similar ranges of imposed force. All data presented in the figures are from *Carausius* and results on *Medauroidea* and summary data on both species are contained in Fig. 9.

### Sensory Recordings

The techniques used to record activities of stick insect tibial campaniform sensilla are described in previous publications (15, 16). Briefly, animals were first restrained on a platform using staples. All nerves to the middle leg were then severed in the thorax. The femur of the middle leg was immobilized using staples and cyanoacrylate adhesive so that the plane of movement of the femorotibial joint was horizontal, parallel to the upper surface of the platform (Fig. 1*D*). The femorotibial joint was also immobilized using a pin and an adhesive. The tarsus was amputated in the distal tibia, near the tibiotarsal joint, and a mixture of Vaseline and paraffin oil was placed over the end of the tibia to prevent desiccation. In most studies, two 50-micron silver wires (Goodfellow Ltd, AG005825) were implanted along the main leg nerve (nervus cruris) in the femur. The insulating layer surrounding the wires was removed over the portion inserted into the leg. Forces were applied to the tibia (see *Mechanical Stimulation*) when positioning the wires, to optimize signals from sensilla with small extracellular amplitudes. The wires were fixed to the cuticle with cyanoacrylate. Sensory activities were recorded using a custom built AC amplifier (Michael Duebber, University of Cologne) and Spike2 interface [Cambridge Electronic Design (CED), Cambridge, UK]. All neurophysiological and force data were recorded using the CED laboratory interface.

### Mechanical Stimulation

Forces were imposed on the tibia using a probe with strain gauges mounted on a motor (Fig. 1*D*; Michael Dȕbbert, University of Cologne). Torque waveforms were derived from a previous study which measured ground reaction forces in freely walking animals via force plates (19). In that study, animals walked on a long narrow platform in which three-dimensional force transducers were embedded. Body and leg motions were captured and reconstructed using a marker-based motion capture system with high-speed cameras (Vicon MX10 with 8 T10 cameras, controlled by software NEXUS v. 1.4.1; Vicon, Oxford, UK). Data from the force plates was low-pass filtered at 12.5 or 25 Hz. Torques about the intrinsic leg joints were determined by inverse dynamics using a three-dimensional rigid link model of the leg, and mean torques were calculated from pooled data.

To study the variable torques of the femorotibial joint of the middle (mesothoracic) leg, we used torque waveforms of individual steps that had maximum values in stance in the directions of flexion or extension [*n* = 8 steps, each derived from a separate walking sequence from *N* = 2 animals (although other animals showed similar variability)]. To use the torque waveforms as stimuli in the present study, torque values were imported into sequencer files in the CED software, and the stance duration was normalized to 800 ms (a typical stance duration for free walking stick insects, Ref. 19). The sequencer files were output as voltages using the CED interface and rerecorded in hardware with low-pass filters to smooth voltage “steps” in the file output. The recorded files were then applied through the motor, which induced torques by bending the tibia around the immobilized femoral-tibial joint. The torque waveforms of single steps (*steps 1–4*, extensors torques; *steps 5–8*, flexor torques) were applied repetitively (mean 0.5 repetitions per second ± 0.004 SD). In this paper, a single repetition of the torque waveform is referred to as a “step” or test. Sensory discharge frequencies were stable and showed no long-term adaptation throughout a series of tests.

### Unit Identification

#### Subgroup identification by cap ablation.

The tibial campaniform sensilla are located on the dorsal (outer) surface of the cuticle of the proximal tibia (Fig. 1, *B* and *C*) and are arranged as two spatially separated subgroups, 6A and 6B (26). The subgroups respond to different directions of forces generated by tibial muscles or applied to the tibia (6B discharges to resisted flexor muscle contractions or applied distal tibial extension; 6A responds to resisted extensor muscle contractions or applied tibial flexion). Units could be identified by the size of their extracellularly recorded amplitude. In the present experiments, bending forces were applied to the distal tibia during placement of the recording wires with the goal of maximizing the recorded amplitude of the smallest 6B sensilla. We used ablation of the subgroups to confirm unit identification: after ablation of the caps of 6B sensilla, subsequent retesting to torque waveforms showed that discharges of 6A sensilla were retained due to their proximal location in the tibia (see *Femorotibial Joint Torques Most Closely Reflect Vertical Ground Reaction Forces* for details). Subsequent ablation of 6A receptors eliminated those components of responses.

Although the tibial sensilla could be analyzed as subgroups, there were limitations to the identification of single units and analysis of frequencies in the multiunit recordings. Responses were recorded through 35–50 repetitions for each step waveform in both the directions of flexion and extension. The size of the recorded potentials decreased somewhat through repeated application of forces to the tibia at different amplitudes. For example, in *Carausius*, the size of potentials of large 6A sensilla decreased, on average, by 18.0% ± 10 SD (*N* = 5) through the tests and subsequent ablation of group 6B sensilla. These decreases were generally proportional in all subgroups and most probably resulted from slight changes in location of the electrodes relative to the main leg nerve. The basic responses of large amplitude 6A and 6B sensilla remained constant, but these changes limited the analysis of 6B small sensilla. We, therefore, selected four to five experiments in each species, in which recordings were the most stable and identification by spike amplitude the most reliable through the entire repeated application of the torque stimuli. However, unit identification was also limited by spike superposition at higher rates and amplitudes of force application (see discussion, *Limitations to the Methods*).

### Morphology

Scanning electron micrographs were taken of the isolated tibiae of the middle legs of newly molted animals using a Hitachi S450 microscope (techniques in Ref. 15).

### Data Storage and Analysis

Data on firing rates and forces were analyzed using Spike 2 scripts. Action potentials were identified as small or large amplitude using CED Spike 2 software functions (sorting or by custom window discriminator scripts). For further analysis, spike frequencies and forces were resampled in bins of 20-, 10-, or 5-ms duration. The highest sampling frequency was used to plot and calculate the latencies of onset of sensory bursts versus force. For plots, the rate of change of force was calculated directly from the recorded force (without filtering) in Excel software. Statistical tests were performed and plotted in SigmaPlot (Systat software) and SPSS (IBM).

## RESULTS

### Variability in Femorotibial Joint Torques Is Not Reflected in Comparable Variability in Femorotibial Joint Angles

**Figure 2.**
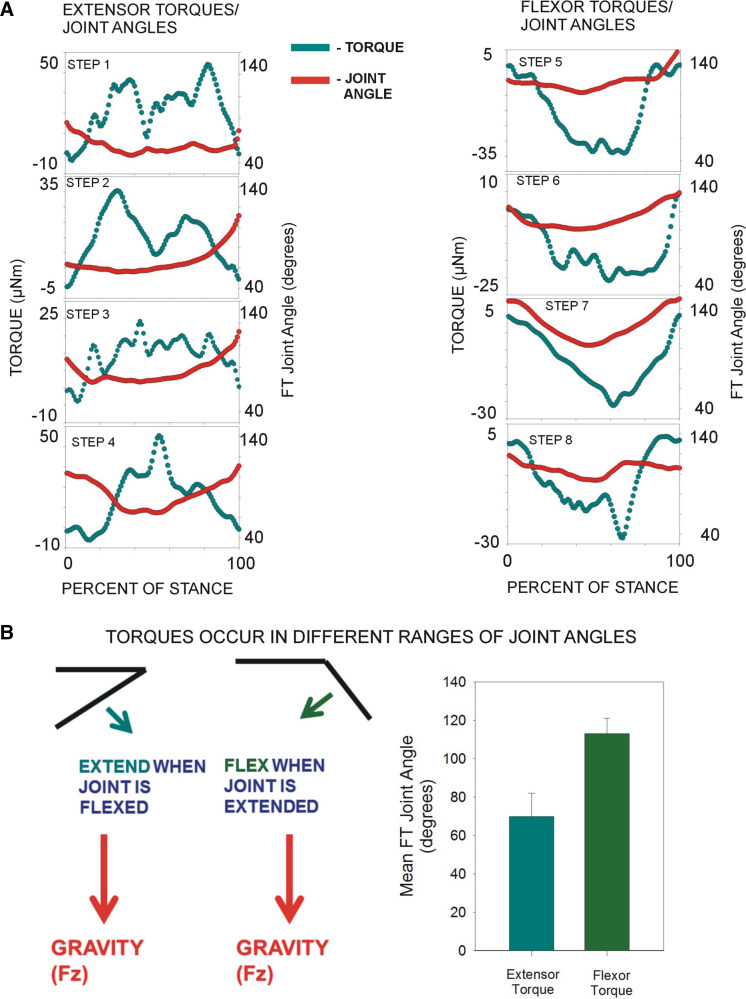
Variability in joint torques and joint angles in single steps. *A*: plots of torques (blue) and femorotibial (FT) joint angles (red) in freely moving stick insects for each of the steps used in the present study. Variability in torques within a step was not reflected in comparable variations in joint angles (although small parallel inflections could occur). *B*: flexor and extensor torques tended to occur in different ranges of joint angles. Consistent with the findings of Dallmann et al. (19), joint torque direction was generally related to the orientation of the tibia. Histogram (*right*) of mean joint angles in which extensor and flexor torques occurred. Extensor torques tended to occur in ranges of joint flexion (<90°) and flexor torques in ranges of joint extension (>90°) (all: *n* = 8 torque waveforms from *N* = 2 animals). Fz, vertical ground reaction force.

In walking stick insects, torques at the femorotibial joint are quite variable and can show changes in sign (flexor vs. extensor; Fig. 1, *E* and *F*). These variations in torques could potentially be associated with fluctuations in the femorotibial joint angle, which can occur in free walking stick insects (27, 28). Figure 2*A* shows plots of the joint torques and femorotibial joint angles for the stance phase of each of the steps examined in this study. In many steps, changes in joint torque were not associated with variations in joint angle, even when the changes in torque were >50% of the maximum value (e.g., *step 2*). In some steps, variations in torque values were accompanied by parallel changes in femorotibial joint angles (e.g., *step 8*), but the magnitude of the angular deviation was usually small in comparison with the size of torque fluctuations.

Although variations in torques within the stance phase were not associated with comparable changes in femorotibial joint angle, we did find that the directions of joint torques (flexion vs. extension) were associated with different ranges of joint angles. Figure 2*B* is a histogram that plots the mean femorotibial joint angle for steps with net extensor and net flexor torques. Flexor torques tended to occur in steps in which the mean joint angle was in ranges of extension (>90°), whereas steps with extensor torques tended to occur in ranges of joint flexion (<90°). The joint torques would, therefore, act to oppose the effects of body weight on the leg (see diagram in Fig. 2*B*).

### Femorotibial Joint Torques Most Closely Reflect Vertical Ground Reaction Forces

**Figure 3.**
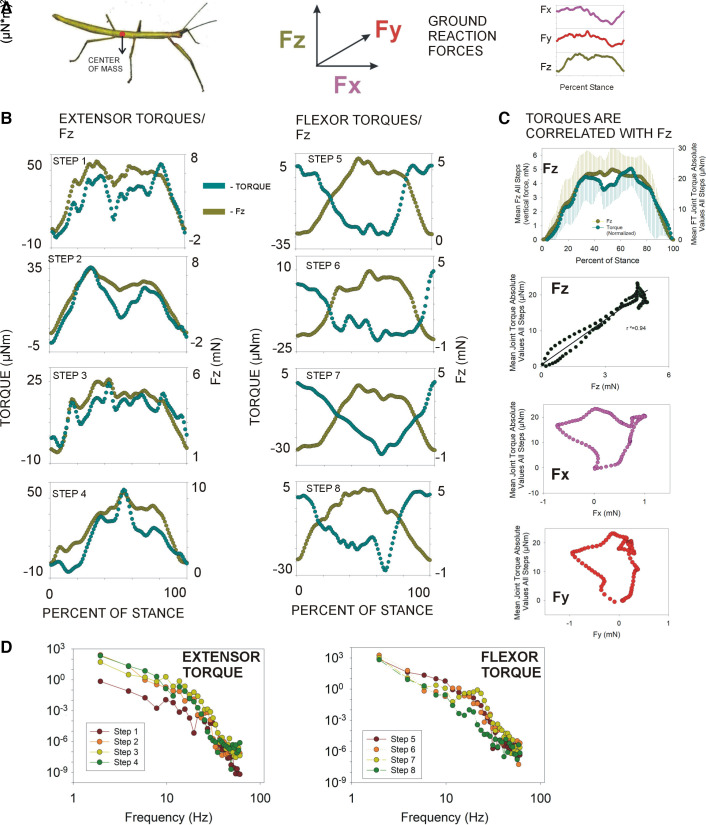
Variability in joint torques and ground reaction forces in single steps. *A*: ground reaction forces—the study of Dallmann et al. (19) characterized the ground reaction forces (Fz vertical, Fx horizontal forward, Fy horizontal transverse) when insects stepped on a force plate. *B*: joint torques and Fz forces in individual steps—plots of joint torques and Fz ground reaction forces from individual steps show similar patterns in many steps. *C*: correlation of joint torques and ground reaction forces—to compare the effects of different ground reaction forces, joint torques were normalized (all positive) and averaged. Plot of the mean joint torque and mean Fz force (*upper left*) during the stance phase show similar profiles. Plots of the magnitudes of mean joint torques vs. mean ground reaction forces show a stronger correlation for Fz (*upper right*) than for Fx (*lower left*) or Fy (*lower right*). *D*: power spectra of joint torques—power spectra (Fourier transform) of extensor (*left*) and flexor (*right*) torques of individual steps were generated in Spike 2 software. Waveforms showed a predominance of slow frequencies with some inflections at moderate rates (all: *n* = 8 torque waveforms from *N* = 2 animals).

When stick insects walk on a horizontal substrate, torques at the femorotibial joint not only provide support of body weight but also generate braking and propulsive forces, as well as providing lateral stability (19). We examined whether the femorotibial joint torques were associated with specific ground reactions forces (Fig. 3*A*). The highest correlation was found between the femorotibial joint torques and the vertical ground reaction forces (Fz). Figure 3*B* contains plots of the femorotibial joint torques and the vertical forces exerted on the force plate for each of the eight steps examined in the present study and shows the general similarity in time course and magnitude of the torques and Fz forces. Figure 3*C* (*upper left*) is a plot of the averaged torques in all steps (normalized for direction) and the Fz force and shows that the relationship was consistent and independent of the torque direction. To further quantify the effects, we plotted the average magnitude of normalized joint torques versus the average ground reaction forces for each direction (Fig. 3*C*; Fz, *upper right*; Fx, *lower left*; Fy, *lower right*). The vertical force (Fz) showed the highest correlation (linear regression, *r*^2^ = 0.94), whereas the relationship with other forces was complex and no direct correlation was apparent (Fx, *r*^2^ = 0.013; Fy, *r*^2^ = 0.001). Figure 3*D* contains power spectra (Fourier transforms generated in Spike 2 software) of the waveforms of the individual steps and shows that, although slow frequencies predominate, there are inflections from the combined effects of these forces at moderate rates. These data support the idea that the forces at the femorotibial joint resist the effects of gravity, depending on the position of the tibia, although transient variations in torque could be associated with forces in other directions (see also Ref. 19).

### Characteristics of Sensory Discharges to Flexion Torques

**Figure 4.**
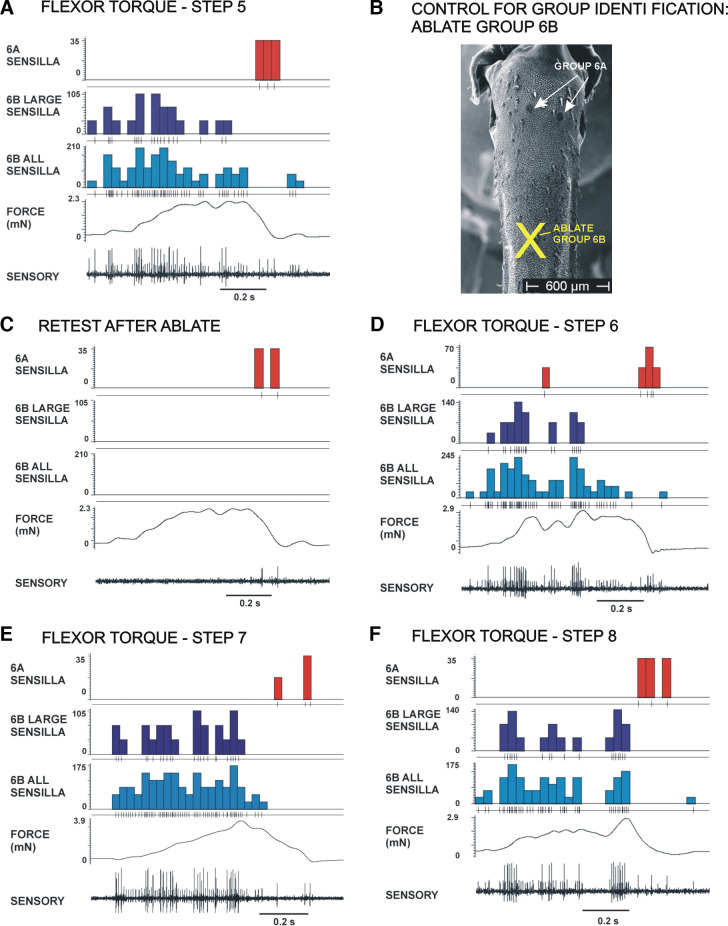
Sensory discharges to flexion torques and controls for group identification. *A*: activities of tibial campaniform sensilla during application of forces using flexion torque waveforms. All recordings showed that multiple units were activated: 6B sensilla discharged during the rising phase of the stimulus. Larger and smaller units could consistently be identified. 6A receptors mainly discharged during the period of slow decline of the stimulus. *B*: control for identification of sensory units. The sensilla of group 6B receptors were ablated without damaging 6A receptors, as the two subgroups are spatially separated (SEM of tibia). *C*: application of torque waveforms after ablation of 6B sensilla showed that discharges of 6B sensilla were eliminated, whereas 6A receptors continued to discharge to torque decreases. Subsequent ablation of 6A sensilla eliminated all recorded sensory activities (data not shown). *D*–*F*: firing of 6B large sensilla occurred in discrete bursts in all steps. SEM, scanning electron micrograph.

Application of torque waveforms was highly effective in activating the tibial campaniform sensilla. Figure 4 shows recordings of sensory discharges to each of the flexor torque waveforms used in the present study (*steps 5–8*, flexion torques). In all tests, forces were applied as dorsal bending of the distal tibia (as occurs to resisted contractions of the tibial flexor muscle) with movement resisted at the femorotibial joint. Sensory discharges were discontinuous and often occurred as three to four bursts in a single torque test (overall stimulus duration was 0.8 s). Sensory responses in the subgroups of sensilla were strictly directional: 6B sensilla (large amplitude units, dark blue histograms; all units, light blue) responded to increases in flexor torques in all animals (*N* = 30 animals), whereas 6A receptors (red histogram) fired, most often, only during the final period of force decline, although single action potentials could occur to some transient force decreases.

Identification of activities as being derived from subgroups 6A or 6B was determined by selective ablation of the sensillum cap (Fig. 4, *B* and *C*). The tibial subgroups in stick insects are spatially separated, permitting ablation of the more distal 6B subgroup without damage to 6A receptors. Tests applied after ablation confirmed that 6B discharges had been eliminated, whereas firing of the 6A receptors persisted, but could be eliminated by subsequent cap ablations in the proximal tibia.

In many recordings, units of larger and smaller amplitude could be distinguished in discharges of 6B receptors, although discrimination of units according to extracellularly recorded amplitude was not consistently possible for 6A sensilla. The larger 6B units did not uniformly increase their discharge frequency during the initial rising phases of torque waveforms but fired in bursts that followed the small fluctuations in force level (Fig. 4, *D* and *F*), even during waveforms that gradually and continuously increased (resembling prolonged ramp functions, Fig. 4*E*). In addition, intense increases in firing occurred to larger transient force increases even after forces had reached maximum levels and the sensory discharges had apparently adapted to the sustained force (Fig. 4*F*). However, transient force decreases produced complete inhibition of sensillum firing, even when they occurred during periods in which forces were generally increasing to high sustained levels (Fig. 4, *A* and *D*).

Analysis of smaller 6B sensory units was limited by spike overlap with larger units in the multiunit recordings. Activities of those sensilla were studied by evaluating all sensory activities above a threshold set to the amplitude of small units. Plots of firing of all sensilla (light blue histograms in Fig. 4) showed more sustained activities than seen in large sensilla. However, sensory discharges did not simply reflect force levels and often ceased during transient force decreases.

**Figure 5.**
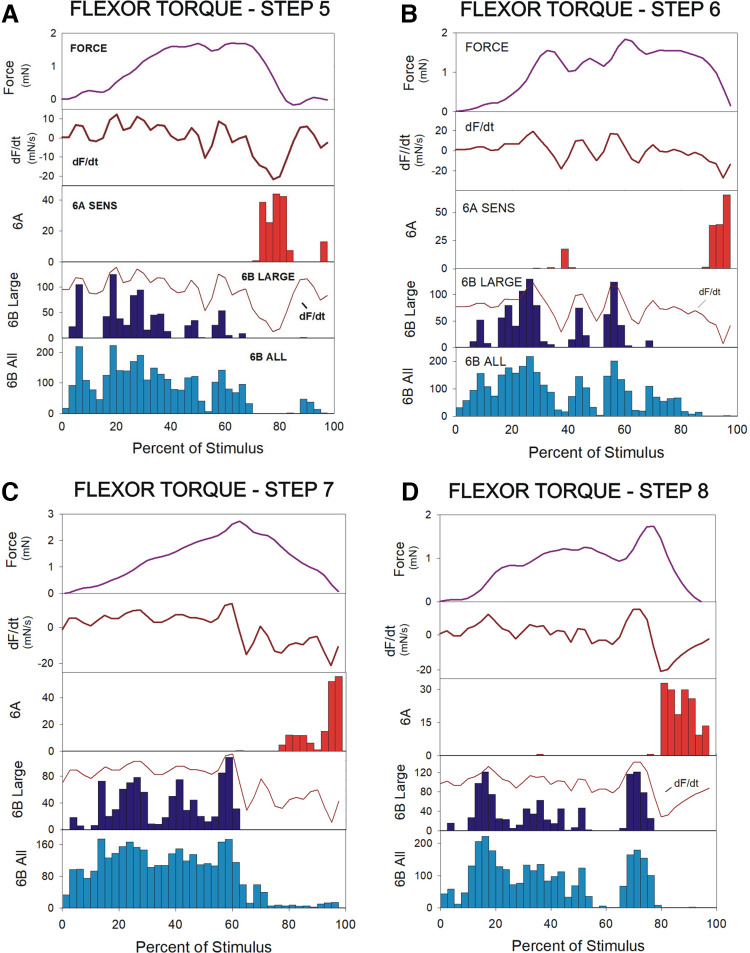
Pooled data: sensory discharges reflect dF/dt in all tests using flexion torques as stimuli. *A*–*D*: plots of pooled data for individual steps. In each plot, the histograms show the mean frequency of sensory discharges (all 6B sensilla, light blue; large 6B sensilla, dark blue; 6A sensilla red). The upper plot shows the mean force and the trace below is the rate of change of force (dF/dt) calculated from the force. The plot of dF/dt is also overlaid on the histogram of the large 6B sensilla, to indicate the correlation between sensory firing and fluctuations in the rate of change of force. [*N* = 4 animals, total number of tests (presentations of stimulus): *n* = 603 in; *A*, *step 6*, 158 tests; *B*, *step 7*, 138 tests; *C*, *step 8*, 181 tests; *D*, *step 5*, 126 tests]. SENS, sensilla.

Analysis of pooled data clearly indicated the relationship between sensillum firing and the rate of change of force (dF/dt). Figure 5 contains histograms of the mean firing of all sensilla, the mean force, and the rate of change of force (as calculated from the mean force measured during the tests) for each of the torque profiles (number of tests *n* = total 603 in *N* = 4 animals; *A*, *step 6*, 158 tests; *B*, *step 7*, 138 tests; *C*, *step 8*, 181 tests; *D*, *step 5*, 126 tests). For clarity, the rate of change of force is also overlaid on the histogram of 6B large firing. In all tests, the peaks in bursts of 6B sensilla indicated increases in the rate of change of force. Firing of 6B large sensilla ceased when the rate of change of force declined, even transiently.

### Sensory Discharges to the Mean Femorotibial Joint Torque and Extensor Torques of Individual Steps

**Figure 6.**
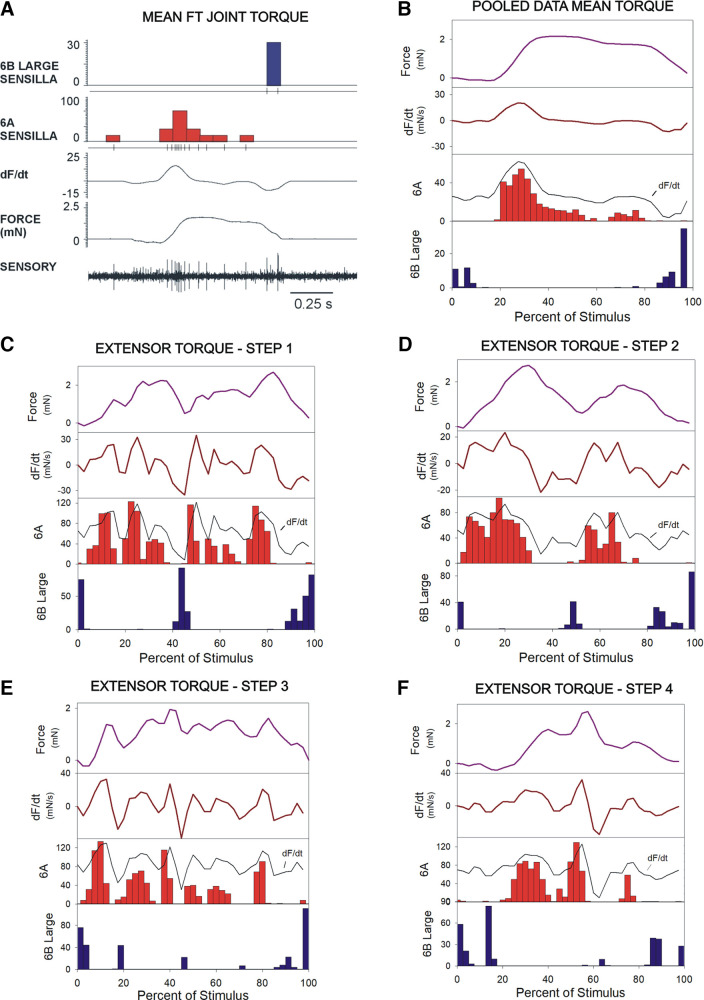
Encoding of mean femorotibial joint torque and torques of individual steps. *A*: the waveform of mean joint torque (see Fig. 1*E*) was applied to the distal femur while recording activities of the tibial campaniform sensilla. *B*: pooled responses to mean torques—group 6A sensilla fired at very low level to the torque increase and 6B receptors discharge when torques declined. *C*–*F*: application of waveforms of individual steps with extensor torques—plots of pooled data from recordings of tests in which extensor torque waveforms of individual steps were applied. 6A sensilla discharged vigorously during periods of force increase, whereas 6B sensilla fired during large or rapid force decreases. 6A sensilla closely followed the rate of change of force (dF/d*t*; traces as in Fig. 5) and did not simply reflect the force level. (*B*: mean torque *N* = 3, *n* = 199 tests; *C*–*F*: *N* = 3 animals, total number of tests *n* = 609; *C*, *step 1*, 134 tests; *D*, *step 2*, 153 tests; *E*, *step 3*, 144 tests; *F*, *step 4*, 178 tests).

Responses of tibial campaniform sensilla were tested using application of the calculated mean femorotibial joint torque, which showed a small net value in extension (19; Fig. 1*E* this paper), as well as individual steps (*steps 1–4*) that occurred as extensor torques that varied from the mean. Figure 6*A* shows a sample recording of activities of tibial campaniform sensilla, and Fig. 6*B* shows pooled responses (*n* = 199 tests, *N* = 3 animals) to application of the mean torque. Figure 6, *C*–*F*, shows pooled data for different individual steps (total number of tests, *n* = 609, *N* = 3 animals; *C*, *step 1*, 134 tests; *D*, *step 2*, 153 tests; *E*, *step 3*, 144 tests; *F*, *step 4*, 178 tests). Responses of subgroups to all extensor torques were directional but opposite to that seen for flexor torques: group 6A sensilla discharged to the torque increase in the direction of extension and 6B receptors fired when the torques decreased. Firing of 6A receptors did not simply reflect the force level but was highly sensitive to increases in the rate of change of force. Although application of the mean torque waveform produced a simple discharge pattern that was maximum after the onset of force application and then declined, firing in single steps occurred as repetitive bursts that were associated with small or large transient increases in the rate of change of force (Fig. 6, *C*–*F*). Discharges ceased during periods of decrease of small (Fig. 6, *C* and *E*) and larger magnitude (Fig. 6, *D* and *F*) even when they occurred during sustained levels of force. Group 6B receptors discharged to the force decline at the end of the stimulus but firing occurred to transient force decreases that were sufficiently large (Fig. 6*D*) or rapid (Fig. 6*C*). Thus, utilization of torque waveforms from single steps showed that the sensilla could monitor changes in forces throughout the stance phase and vigorous discharges were not merely limited to the period immediately following stimulus (stance) onset, as was elicited by application of the calculated mean torque.

### Sensory Discharges Encode the Rate of Change of Force Increases but Firing Is Suppressed during Transient Force Decreases

**Figure 7.**
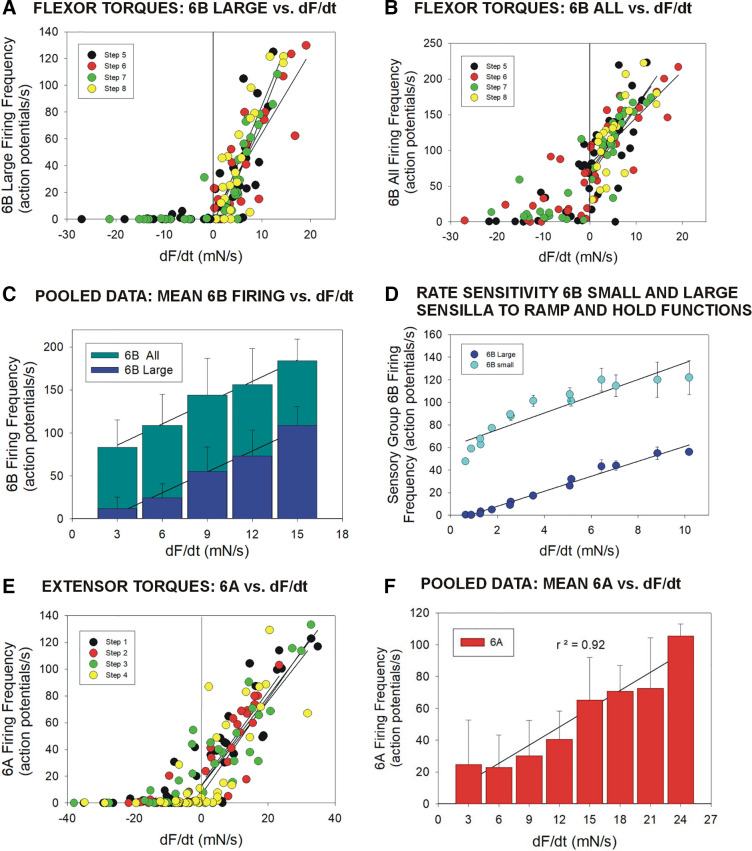
Sensilla encode positive values of dF/dt. *A* and *B*: plots of firing frequencies of 6B large sensilla (*A*) and all 6B sensilla (*B*) vs dF/dt in tests of application of flexor torques (same dataset as Fig. 5; *n* = 603 tests, *N* = 4 animals). Mean values of individual steps are coded by color in each plot. Positive values of dF/dt are on the *right*, negative values (force decreases) are on the *left*. Receptors discharged vigorously to force increases, but firing was significantly decreased during transient force decrements despite the sustained high levels of force. The regression lines were calculated for dF/dt values > 0 for each test. *C*: pooled data of 6B firing. The histogram plots the mean firing frequency of 6B large (dark blue) and all 6B (light blue) vs dF/dt in all tests of flexor torques. 6B large sensilla are highly rate sensitive, and the addition of smaller receptors increases the firing frequency but does not change the slope (slope: 6B large = 8.09; 6B all = 8.28). *D*: rate sensitivity to application of ramp and hold functions of varying rates of rise. Within the range tested, the rate sensitivity to constant rates of increase is similar to that seen in tests using the variable waveforms of joint torques (*n* = 10 repetitions of 7 rates in *N* =1 animals, see also Ref. 15, Fig. 7). *E*: plot of pooled data of 6A firing frequencies during application of extensor torques similar to *A* and *B*. *F*: histogram of mean firing of 6A sensilla during all tests (*n* = 609 tests, *N* = 3 animals). dF/dt, rate of change of force.

Data on discharges of campaniform sensilla to torque waveforms were pooled and analyzed to evaluate the consistency of encoding of dF/dt. Figure 7, *A* and *B*, plots the mean firing frequencies of 6B large (Fig. 7*A*) and all 6B (Fig. 7*B*) sensilla at different rates of change of force (flexion torques). These data are derived from the same tests as Figs. 5 and 6 (*n* = 603 tests, total *N* = 4 animals). Each point represents the mean value (sampling interval 20 ms) of firing, and rate of change of force and individual steps are indicated in different colors. These plots indicate some dispersion of values, but discharges of 6B sensilla occur predominantly during the periods of increasing force (positive dF/dt), whereas firing is significantly decreased or ceases completely during force decreases. Discharges during intervals of force decreases are more frequent when smaller units are included (Fig. 7*B*), consistent with their higher tonic sensitivity. Figure 7*C* is a histogram of pooled data of 6B discharges from all tests showing that there is strong overall correlation between sensillum firing and dF/dt (regression calculated for positive values of dF/dt: *r*^2^ = 0.98 for 6B large and all 6B sensilla). Furthermore, the effect of inclusion of smaller units is to increase the discharge frequency of the group as a whole without changing the relative sensitivity to force dynamics (6B large slope = 8.1, 6B all slope = 8.2). These sensitivities of 6B receptors to the rate of change of force are also indicated in tests of using ramp and hold functions (Fig. 7*D*; see also Ref. 15): firing of smaller units reflects the rate of change at very low forces but responses begin to saturate at moderate rates of dF/dt. Figure 7, *E* and *F*, shows plots of firing of group 6A sensilla at different rates of change of force from individual tests (Fig. 7*E*) and pooled data (Fig. 7*F*) from tests of application of extensor torques (*n* = 609 tests, *N* = 3 animals). Receptor discharges again occur predominantly during periods of force increase and firing is greatly reduced by transient force decreases (regression calculated for positive dF/dt: *r*^2^ = 0.92). These data indicate that signals from campaniform sensilla are strongly correlated with the rate of change of torque increases that could occur during walking.

### Force Encoding of Amplitude Is History Dependent and Shows Hysteresis

**Figure 8.**
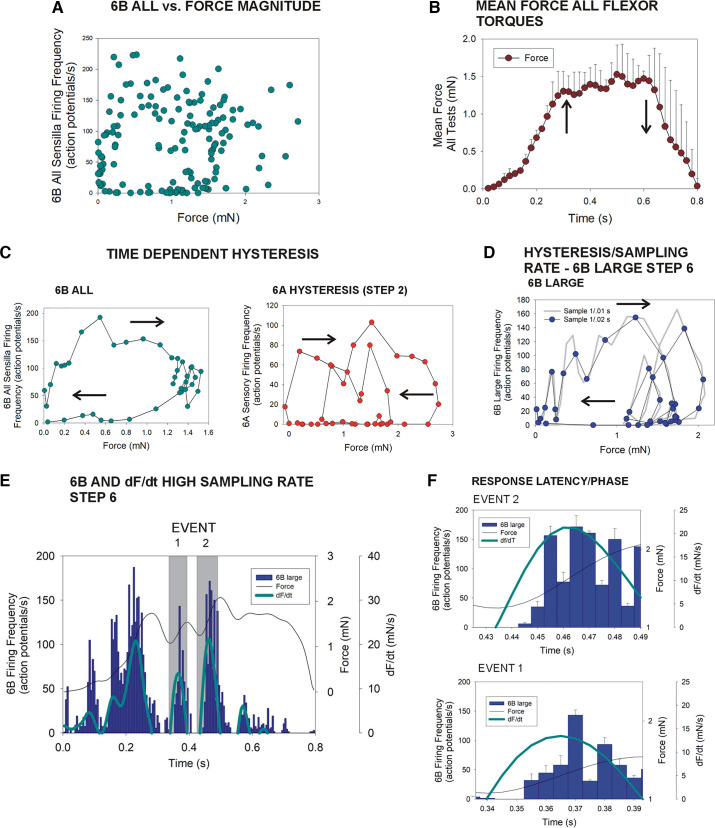
Time-dependent hysteresis and response latencies. *A*: plot of the firing frequencies of all 6B campaniform sensilla vs. level of applied force for all tests applying flexor torques: there is no linear correlation, although the plot forms an apparent ring. *B*: mean change in force level of all flexor torques over time. *C*: time dependence—mean firing was calculated in the sequence of occurrence in tests of flexor torques for all 6B sensilla (*left*) and 6A receptors in tests of *step 2* extensor torques (arrows indicate temporal sequence). Mean firing shows substantial time-dependent hysteresis. Discharges of 6A sensilla show two loops as torques in *step 2* initially rose, then declined, and subsequently increased again. *D*: similar plot of 6B large receptors shows a hysteresis loop at two sampling rates (bin 0.02 s, black line; bin 0.01, gray line). *E*: plot of discharges of 6B large sensilla sampled at a high rate (bin = 0.05 s). Bursts are closely correlated with positive values of dF/dt (light blue line) that correspond to increases in force (black line). *F*: response latency—the high sampling rate was used to determine the latency of 6B firing during the rising phases of dF/dt (marked by gray shading in *D*). Sensillum firing was initiated during the rising phase of dF/dt in all tests (*A*, *B*, *C left*, *D*: *N* = 4 animals, *n* = 603 tests; *C*, *right*: *N* = 3 animals, *n* = 609 tests; *E*, *F*: *N* = 5 animals, *n* = 166 tests). dF/dt, rate of change of force.

Data from all tests were also analyzed to determine the effects of force magnitude on sensillum discharges. Figure 8*A* plots the firing frequencies of all 6B sensilla versus force magnitude for stimuli that mimicked flexion torques. There is no linear correlation but the data appear to generally form a ring. We, therefore, analyzed the relationship between sensillum firing and force level as a temporal sequence. Figure 8*B* shows that the mean force that was applied in all tests of flexor torques first increased, then varied, and finally declined. Plots of the mean firing frequencies of all 6B sensilla versus force in the sequence of their occurrence in tests (Fig. 8*C*, *left*) formed rings indicative of hysteresis in encoding of force level. Similar analysis of responses of 6A sensilla to extension torques (Fig. 8*C*, *right*) also showed hysteresis rings, but data for some steps (e.g., shown *step 2*) were complicated by the larger variations in torques, which could rise and decline repeatedly within a test, producing multiple hysteretic rings. Figure 8*D* is a similar plot for 6B large sensilla with data sampled at two intervals (0.02- and 0.01-s intervals) that showed similar hysteresis at both sampling rates. We also examined the effect of sampling data at higher rates (0.005-s bins, Fig. 8, *E* and *F*), which permitted analysis of the latency of onset of discrete sensory bursts. Sensillum firing was initiated during the rising phase of dF/dt in all tests at short latency (mean latency = 14.5 ms ± 2.8, *N* = 5 animals, *n* = 312 bursts).

### Responses of Tibial Campaniform Sensilla in Medauroidea Stick Insects

**Figure 9.**
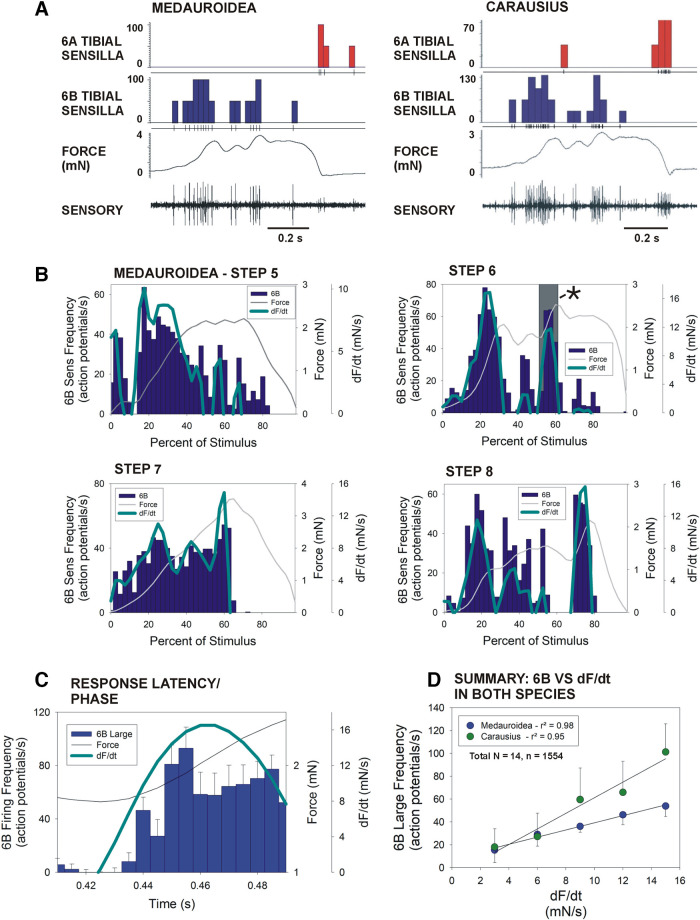
Responses of tibial campaniform sensilla in middle legs of *Medauroidea* stick insects. *A*: sensory discharges to torque waveforms. Recordings of tibial campaniform sensilla of middle legs in *Medauroidea* (*left*) and *Carausius* (*right*) stick insects to application of the flexor torque waveform of *step 6*. In both species, group 6B receptors fired in bursts to torque increases, whereas 6A sensilla discharged to large torque decreases. Larger forces were required to elicit discharges in *Medauroidea* in comparison with *Carausius* (note difference in force scale), consistent with their larger size and mass. *B*: pooled data of responses in *Medauroidea*. Histograms of firing frequencies of 6B sensilla (dark blue bars) with overlaid line plot of dF/dt (light blue, scaled to show only positive values) and force magnitude (black lines). As in *Carausius*, *Medauroidea* 6B sensilla fired in bursts that were closely correlated with positive values of dF/dt. *C*: latency to 6B firing. Data were sampled at a high rate (bin = 5 ms, as in Fig. 8*D*) and periods of discrete bursts were extracted. Histogram of mean sensory discharge (with SD) of 6B sensilla with line plots of dF/dt (light blue), and the force during a period in tests of torque *step 6* (indicated by an * in *B step 6*). The 6B large sensillum bursts are consistently initiated during the rising phase of dF/dt (mean latency 17.5 ms ± 2.8 SD). *D*: summary plot of mean 6B firing frequencies (+SD) in different ranges of dF/dt. Discharges of large sensilla in *Medauroidea* and *Carausius* encode the rate of change of force (regression *r*^2^ = 0.98, *Medauroidea*; *r*^2^ = 0.95, *Carausius*) but the slopes differ (*Medauroidea*, slope = 3.13; *Carausius*, slope = 6.83) (*B*: all plots, *N* = 4 animals; *step 5*, *n* = 165 tests; *step 6*, *n* = 186 tests; *step 7*, *n* = 165 tests; *step 8*, *n* = 196 tests; *C*: *N* = 4 animals, *n* = 186 tests; *D*: *N* = 14 animals, *n* = 1,554 tests). dF/dt, rate of change of force.

Activities of tibial campaniform sensilla in middle legs of *Medauroidea* stick insects were also recorded to application of the torque waveforms (Fig. 9). Despite the difference in their size and mass (25), similar patterns of discharge were seen in both the species (Fig. 9*A*), although response frequencies were lower in 6B large sensilla in *Medauroidea* than in *Carausius* to equivalent forces (6B small sensilla could not be consistently analyzed in *Medauroidea*). Application of waveforms of flexor torques of single steps (Fig. 9*B*) also showed similar patterns of bursting that closely reflected the rate of change of force (dF/dt). Measurements of the latencies of burst onsets to increases in dF/dt (from pooled data sampled at a high rate, bin 0.005 s) demonstrated that 6B sensory discharges were initiated during the rise phase of force at relatively short latency (mean 17.5 ± 2.8 ms, *N* = 4 animals; *n* = 186 tests). Figure 9*D* is a summary plot of pooled data of discharges of 6B large sensilla to all imposed flexor torques in both species. Firing frequencies reflected the rate of change of force, but the slope (gain) was higher in *Carausius* than in *Medauroidea*. Current experiments are examining whether this difference in sensitivities is correlated with the larger size of *Medauroidea* stick insects.

### Effects of Increasing Force Amplitude on Sensory Encoding of Joint Torques

**Figure 10.**
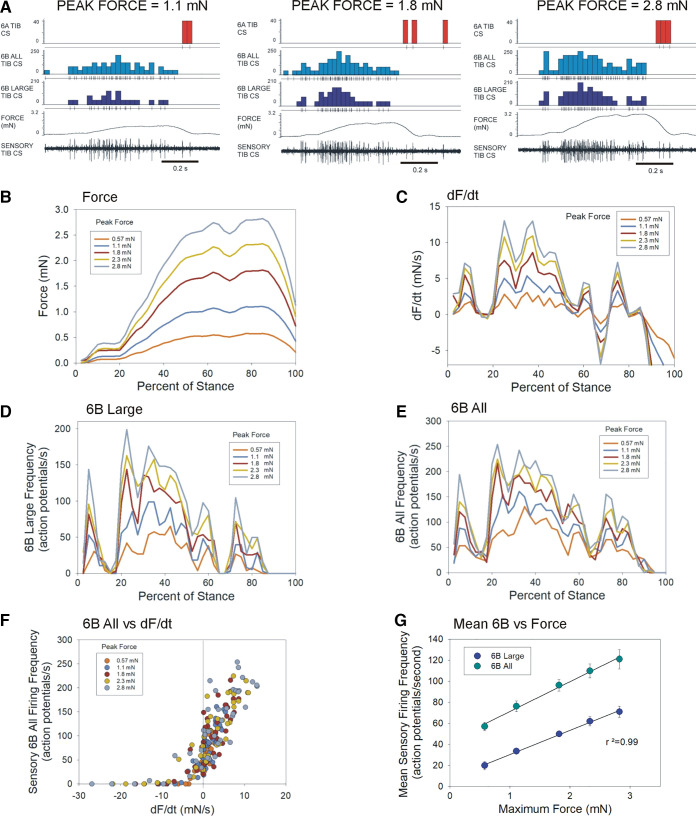
Effects of varying torque magnitude on encoding of force and dF/dt. *A*: the waveform for flexor torque of *step 5* was applied at different amplitudes: recordings of sensory encoding at peak force 1.1 of 2.8 mN (traces as in Fig. 4). Plot of forces (*B*) and rate of change of force (*C*) for torques applied at different maximum amplitudes. *D*: plots of discharge frequencies of 6B large sensilla closely reflected positive values of dF/dt. *E*: plot of discharges of all 6B sensilla. Firing was similarly modulated in all 6B sensilla and addition of smaller sensilla raised the overall firing frequency. *F*: plots of sensory firing of all 6B sensilla vs. dF/dt (similar to Fig. 7*B*). Sensory discharges reflected the rate of change for force increase, but firing was suppressed during periods of force decrease. *G*: plot of mean frequency of firing of 6B large (dark blue) and 6B all sensilla averaged in tests applied at different force levels. The average discharge reflected the magnitude of the force (*D*–*G*: *n* = 115 total, mean 23 repetitions at each level, *N* = 1). dF/dt, rate of change of force; TIB CS, tibial campaniform sensilla.

Insects can use sensory information to make rapid adjustments in walking to loss/damage of a single leg that increases body load supported by the remaining legs (29). We addressed the problem of variable distribution of body load by studying the effect of progressively increasing the magnitude of torque waveforms. Figure 10 shows sensory discharges obtained from application of the flexor torque of *step 5* at different maximum amplitudes (Fig. 10*A*; *n* = 115, *N* = 1 animal, *Carausius*). The peak force (Fig. 10*B*) was progressively increased from 0.57 to 2.8 mN, which also resulted in an increase in the rate of change of force (Fig. 10*C*). As in other tests, discharge frequencies of 6B large sensilla closely reflected increments of dF/dt (Fig. 10*D*). Firing was similarly modulated when sampling included smaller sensilla (Fig. 10*E*), particularly in tests at higher force amplitudes, which also raised the overall firing frequency. Figure 10*F* plots the discharge of all 6B sensilla versus dF/dt in all tests: despite noticeable spread in data at low rates of change, similar sensitivities and encoding were obtained in tests at higher force amplitudes (force/slopes of regression: 0.57 mN/24.3, 1.1 mN/18.3, 1.8 mN/12.3, 2.3 mN/11.2, 2.8 mN/9.9), similar to the findings of previous studies on the effects of force magnitude on rate sensitivity. Figure 10*G* is a plot of the mean firing frequencies averaged over the entire duration of each test. Despite the variability due to sensitivity to the force rate, when averaged over time, the discharges of the campaniform sensilla reflect the overall force magnitude.

### Replicating the Effects of Joint Torques on Adaptation Using Mechanical Stimuli with Exponential Rates of Increase

**Figure 11.**
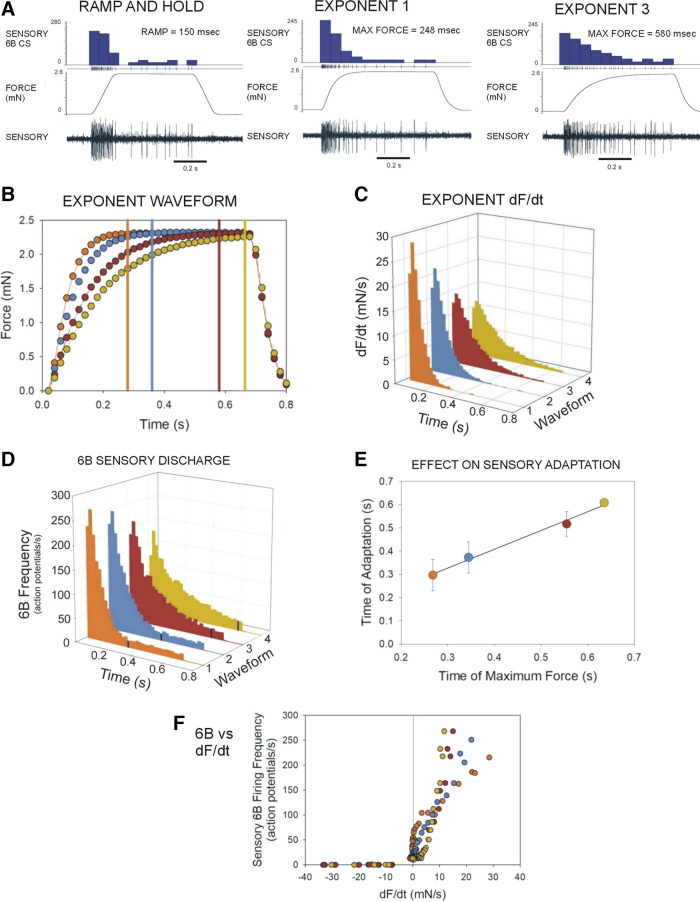
Replicating components of naturalistic stimuli using mechanical stimuli with exponential rates of increase. *A*: recordings of stick insect tibial campaniform sensilla during application of forces using ramp and hold functions (*left*) and waveforms that exponentially approached a maximum (*middle*, *right*). Histograms of 6B large sensilla (blue) show that the initial component of firing is prolonged to exponential functions. *B*: plot of exponential waveforms. Vertical lines indicate time of attainment of maximum force. *C*: plots of F/dt for each of the waveforms in *B*. *D*: plots of 6B firing frequencies to each wave forms. Black lines indicate time of onset of adapted discharge. *E*: plot of time of onset of adapted discharges for each waveform. Adaptation is delayed when positive dF/dt is prolonged. *F*: plot of sensory frequency vs. dF/dt. In all tests, sensilla encode the rate of firing to all positive values of dF/dt. (*C*–*F*: *N* = 3 animals, *n* = 622 tests; in *F*, each point is the mean frequency at that rate). CS, campaniform sensilla; dF/dt, rate of change of force.

Our previous study (18) showed that application of forces using torque waveforms could substantially reduce motor adaptation seen to conventional ramp and hold waveforms. Analysis of torque waveforms suggested that the onset of adaptation was delayed due to the sustained period of increase in dF/dt. To replicate the initial component of the torque waveforms in the present study, we recorded activities of campaniform sensilla while applying waveforms with exponential rates of increase that reach a force maximum after varying times (Fig. 11, *A* and *B*). Figure 11*C* is a plot of the rate of change of force, and Fig. 11*D* is a similar plot of the discharges of 6B large sensilla during these tests (*n* = 622 tests, in *N* = 3 animals). We also examined whether the time of onset of adaptation was related to the time of maximum force. The adapted firing frequency was determined by averaging the sensory firing in the last 25% of the hold phase except waveform 4 (the most gradual rise) which was 15% of the hold phase. The time of adaptation was determined as the temporal occurrence of the first bin equal to that value [that was not followed by an increase in the average value of the succeeding (1–3) bins]. These experiments showed that there was a strong correlation between the time of attainment of peak force (dF/dt = 0) for each waveform and the time of onset of adaptation of the sensory discharges (Fig. 11*E*). Plots of the firing frequency versus dF/dt were similar to those obtained with torque waveforms in showing discharges during the rising phase that immediately ceased when forces declined (Fig. 11*F*). These findings suggest that one effect of torque waveforms is to produce graded changes in force signals that could contribute to the smooth and continuous modulation of motor activities in walking.

## DISCUSSION

The goals of the present study were to characterize encoding of “naturalistic” forces by tibial campaniform sensilla to evaluate the types of information they provide and gain insight into their potential functions in walking. We refer to the stimuli using waveforms of joint torques as “naturalistic” because they are based on measurements made in freely moving animals, in contrast to waveforms used in many studies. We used the torques of the femorotibial joint of stick insects as mechanical stimuli as they showed considerable variability in animals that walked freely on a horizontal substrate, including changing torque direction from extension to flexion. Analysis of kinematics and ground reaction forces showed that the sign (direction) of the torque was related to the angular range, whereas the magnitude of the torque most closely reflected the gravitational vector (Fz) and, potentially, the distribution of body load among the legs. Analysis of sensory discharges confirmed that the system is particularly sensitive to the rate of change of forces and can rapidly signal torque increments. These findings are discussed under *Stabilizing Distal Joints While Forces Are Exerted at Proximal Joints* below in the context of a model in which force detection can aid in adjusting ongoing muscle contractions while the system produces requisite leg movements.

### Joint Torques and Variability of Forces at the Stick Insect Femorotibial Joint

Campaniform sensilla encode strains in the exoskeleton, and some groups of receptors effectively monitor the net forces generated by resisted contractions of groups of muscles acting at a joint, which can be calculated as the joint torque (12, 18, 30). Dallmann et al. (19) measured the ground reaction forces and joint movements in stick insects walking on a horizontal substrate and calculated the torques at the major intrinsic leg joints. That study showed that torques are functionally segregated: the largest torques were generated in support and propulsion at the proximal, coxo-trochanteral joint, whereas smaller torques occurred at the leg joint with the body (thoraco-coxal) and the more distal “knee” (femorotibial, FT) joint.

Although the mean joint torques at the femorotibial joint were relatively small, there was considerable variation both in the sign (direction) and the magnitude of torques, as also occurs in other insects (31) and in the human knee joint in walking (see data of Refs. 32 and 33). Previous studies have shown that the middle legs of insects are used flexibly and can exert “braking” or propulsive force in walking (34, 35), as well as contributing to turning movements (36) and lateral stability (37). Our analysis of steps with the largest variations (in the absence of external perturbations or changes in the direction of progression) showed that, when walking on a horizontal substrate, torque variability within the stance phase was not consistently associated with comparable changes in joint angles. In these steps, parallel inflections could be seen in plots of both variables but the relative magnitude of change in joint torques was much larger than variations in joint angles (Fig. 3), and in many steps, no comparable changes occurred. Relative constancy of kinematics and variability in kinetics also occurs in stick insects walking on inclined surfaces, although the average magnitudes of torques at the femorotibial joint are larger when traversing nonhorizontal surfaces (38). The direction of torques was related to the ranges of joint angles of the femorotibial joint during stance, as flexor torques were generated when the joint angle was in ranges of extension and extensor torques in ranges of joint flexion. These forces would act to stabilize the joint and counter movements toward the extremes of joint angle (19), and similar relationships have been shown in the human knee joint (39, 40).

Evaluation of the ground reaction forces indicated that magnitudes of the femorotibial joint torques in steps with large variations were most consistently correlated with the vertical force (Fz), which reflects the force of gravity and supportive functions of the middle legs on a horizontal substrate (41, 42; humans: Ref. 43). Torques of some individual steps could show variations (e.g., Fig. 3*B*, *step 8*) that were correlated with other vectoral directions, but these effects were not consistent or predominant. The major source of torques in the legs of many animals is derived from the body mass and the distribution of the resultant forces among the legs depends on the gait (34, 44). In slow walking, gaits of many insects are not fixed but based on a metachronal pattern (posterior to anterior sequence of leg lifting) that can result in a variable number of legs providing support (45, 46). The magnitude of the force variations can also depend on walking speed: in more rapid walking and slow running, the pattern approaches a tripod gait in which a middle leg provides all the support of body weight on one side. The correlation between variations in joint torques and vertical ground reaction forces was unexpected given its lateral location and functions in determining walking direction. However, the data on “outlier” steps support the idea that the FT joint acts to stabilize the body to resist the effects of gravity while large forces are exerted (e.g., in propulsion) by muscles acting at the coxo-trochanteral joint (19).

### Sensory Discharges to Joint Torques

The tibial campaniform sensilla responded vigorously to forces imposed on the leg using the profiles of femorotibial joint torques. As in previous studies in restrained preparations (15), responses of the subgroups of receptors were directional: 6B sensilla discharged to force increases in the direction of joint extension and force decreases toward flexion, whereas 6A receptors showed opposite directional sensitivity. The tibial sensilla also encode forces generated by resisted muscle contractions with the same directionality (16). The patterns of activities found in stance in “single leg stepping” were similar to that seen to flexion torques in the present study: 6B sensilla fired to force increases, whereas 6A receptors discharged at the end of stance before leg lifting in swing (16, 47). Similar patterns of activity were also found in the subgroups of tibial sensilla of cockroaches both in free walking animals and in preparations “walking” on slippery surfaces (48). These findings reflect the activation of the flexor muscle early in stance to support body weight in upright walking in both species (49–52; see also Ref. 47 in locusts).

### Use of “Naturalistic” Stimuli Reproduces Characteristics of Sensory Discharges Reflecting Load Transfer among Legs in Freely Walking Insects

A major feature of discharges of tibial campaniform sensilla to “naturalistic” torque stimuli was their sensitivity to fluctuations in forces (53, 54). In many tests of flexor torques, firing of large 6B receptors occurred as a series of bursts, whereas discharges of smaller 6B receptors showed strong modulation of firing frequency. Similarly, 6A sensilla typically discharged in bursts to extensor torques, even at high levels of sustained forces. Analysis of these data showed that sensory firing closely reflected the rate of change of force (dF/dt) and pooled data indicated significant correlation of discharge frequencies and the rate of force application. A number of studies have shown that campaniform sensilla, like Golgi tendon organs of vertebrates, are highly sensitive to and encode force dynamics (55, 56), as are many other mechanoreceptors (57). In addition, another factor that could contribute to sensory “bursting” rather than continuous discharge is the relatively small magnitudes of the femorotibial joint torques. We evaluated the effects of force magnitude by varying amplitude of single torque waveforms: these tests showed that spike rate increased but the dependence on dF/dt persisted, as did cessation of firing during periods of −dF/dt.

Furthermore, discharges of tibial campaniform sensilla to imposed “naturalistic” joint torques showed many characteristics that closely resemble sensory activities recorded in freely walking insects. Although recordings of campaniform sensilla have not been obtained from free walking stick insects (due to technical problems of cross talk and the narrow diameter of leg segments), discharges of tibial sensilla have been extensively recorded in free-moving cockroaches (48, 58, 59). Those recordings show substantial fluctuations in firing frequencies during individual steps in stance and often occur as repetitive bursts within intervening silent periods. This variability was not quantified in previous studies, which did not record ground reaction forces, and it is not apparent in pooled or averaged data but was found in steps in all recorded sequences. In contrast, discharges recorded in stepping with body weight supported (which eliminates or minimizes the effects of load transfer among legs) showed much more uniformity and smaller variations in sensory firing frequency (48). Further studies are planned to quantify the effects of load transfer, but the present findings support the idea that a major source of variability of sensory discharges to joint torques reflects forces exerted by other legs in support and propulsion in free walking animals.

### Importance of dF/dt in Walking: Forces Are Dynamic and There Is No “Static” Hold Phase

Our studies showed that the tibial campaniform sensilla of both larger and smaller amplitude potentials effectively encode the rate of change of force increase (dF/dt) to “naturalistic” torque stimuli. Sensitivity to dF/dt was pronounced in the torque waveforms that were derived from “outlier” steps but also occurred to application of the calculated mean torque waveform. In addition, almost all torque waveforms showed graded variations in force through the stance phase and did not (or only briefly) achieve a “static” level. Thus, the predominance of dynamic sensitivity simply reflects the fact that the forces occurring during walking are changing throughout the stance phase. Some of the variation is “masked” in calculation of the mean torque but is particularly apparent when “naturalistic” waveforms of single steps are applied that retain force variations.

In our previous study, we noted that sustained positive values of dF/dt in joint torques could delay adaptation of motor discharges (18). In the present study, we replicated the initial rising phase of naturalistic torque stimuli using exponential functions that gradually achieved a static level after variable intervals. These tests showed that sensory discharges were elevated for the entire period in which forces were increasing and that there was no discrete or abrupt transition to a tonic level as occurs in tests using ramp and hold functions. Even slow rates of change in exponential functions receptors maintained elevated firing frequencies and delayed the onset of adapted firing when compared with ramp and hold stimuli. Ramp and hold waveforms have been valuable in evaluating and discriminating components of sensory discharges that encode the level or rate of change of the stimulus. However, the functions are discontinuous and can be, in a sense, misleading as they do not accurately replicate the smooth transitions and lack of a completely static hold phase that occur normally in behavior.

Signals of force dynamics are now considered to play an integral role in control of posture and locomotion (11), and regulation of force dynamics may underlie the symptoms of some neurological disorders (e.g., spasticity; Ref. 60). Although dynamic sensitivities might apparently compromise the system’s ability to “calculate” body weight, signals of force are consistently used convergently with other proprioceptive signals in detection and compensation for load (9, 61, 62), in agreement with the early suggestion by Jami (10). Finally, as we noted in tests of torque waveforms applied at different levels, summing the afferent signals over time clearly reflected the force magnitude (the integral of dF/dt is F). Although not extensively studied, filtering and summation of sensory signals may provide the nervous system with an indicator of body mass.

### Influence of Past History and the Control of Skeletal Muscle

In all tests in which forces were applied using naturalistic torque waveforms, campaniform sensilla showed substantial hysteresis in discharge frequency during intervals of force decrease, even when forces were applied at high levels. Periods of complete cessation of firing were seen in sensilla with large extracellular amplitude and in small units that could encode force magnitude as they have tonic discharges even at low amplitudes. Hysteresis has been previously demonstrated in restrained preparations in the tibial campaniform sensilla of stick insects and cockroaches and in trochanteral receptors of the stick insect (62) and has been noted in a number of other sensory systems (vertebrates: Refs. 63 and 64; invertebrates: Refs. 65–69).

The functions of hysteresis in discharges of sense organs are unclear, but they may be adaptive and act to reduce residual tensions in leg muscles (3, 70). A similar role for proprioceptive modulation of muscle tensions was postulated by Nichols and Houk (71) who suggested that “the main function of autogenetic reflexes may be to compensate for variations in the properties of skeletal muscle rather than to oppose changes in load” (71). Many motor neurons show “plateau”-like responses in which discharges persist after excitatory inputs have decreased (72–75). This property can generate persistent muscle tensions, and the precipitous decrease in sensory receptors to decreases in force may serve to counter that effect. These effects may also depend on filtering characteristics of responses in interneuronal encoding of force. In locusts, spiking interneurons show little hysteresis to proprioceptive inputs (76), whereas nonspiking interneurons that may reflect summed discharges, and potentially low-pass filtered versions of sensory discharges, can show substantial hysteresis to proprioceptive inputs (77).

### Limitations to the Methods of This Study

The requisite use of multiunit recording to study encoding of forces at the rates and levels that occur during walking imposed limitations in data analysis of sensory responses (78). Although single units could be discerned by imposing forces at low levels, application of forces at the magnitude of the torques of freely walking animals could produce spike superposition, limiting analysis of spike frequencies at higher rates. Furthermore, measurements of discharge frequency were limited in accuracy by the presence of units with different sensitivities, as well as variations in stiffness of the cuticle (79). Binning of data decreased the effect of anomalous high frequencies when spikes nearly coincided but limited analysis that could determine maximum sensory firing and correlate the phase of peak afferent frequencies with specific parameters of forces (53). Extending analysis to smaller bins (0.005 ms) permitted examination of the latencies of onset of afferent bursts relative to the change in force (Figs. 8 and 9). These limitations could be addressed in future studies if the technical challenges of applying forces at high levels in preparations that permit recording of single sensilla can be overcome. It is also important to note that the goal of our experiments differed from other studies that tested whether sensory encoding of naturalistic stimuli differed from discharges to application of conventional waveforms (53). Our study sought to use naturalistic stimuli to gain insight into the parameters of forces that are signaled in walking of freely moving animals and the potential functions of force feedback. The dynamic and static response properties of campaniform sensilla seen in our experiments are basically similar to many previous studies (17), and the fluctuations in the joint torques most often occurred at relatively slow frequencies. In addition, the complex features of force encoding, such as hysteresis, have recently been modeled in a study that reproduced many aspects of force transduction by campaniform sensilla (24).

### Stabilizing Distal Joints While Forces Are Exerted at Proximal Joints

Our data support the idea that discharges of the tibial campaniform sensilla can aid in stabilizing the femorotibial joint in walking on a horizontal substrate, as proposed by Dallmann et al. (19). Although the effects of forces on motor outputs have not yet been directly tested using torque waveforms in stick insects, previous studies have demonstrated that tibial campaniform sensilla have similar effects on motor outputs in a number of insect species. In stick insects, punctate stimulation of cuticular caps of individual 6B sensilla excited the slow tibial extensor motor neuron, whereas stimulation of 6A receptors inhibited extensor firing (15). These effects on extensor motor neurons in stick insects have been confirmed using bending forces applied to the tibia (80). The same effects on extensor firing were found by cap stimulation of the subgroups of tibial campaniform sensilla in cockroaches, which also demonstrated reciprocal influences on activities of the tibial flexor muscle (81), as has been reported for locust tibial campaniform sensilla (47).

These effects of sensillum activation could dynamically adjust motor outputs to counter the torque variations: flexor torques that excite 6B receptors should increase the probability of activation of the extensor, whereas extensor torques would tend to inhibit extensor and potentially activate flexor motor neurons. The effect of sensillum firing would be to enhance the activities of muscles to stabilize the joint (82). In cockroaches, these effects were consistent with the patterns of motor activation seen in walking: flexor muscle firing after stance onset elicited discharges of the proximal tibial sensilla which enhance extensor firing (48, 58). In addition, although the effects of force inputs at the coxo-trochanteral have been shown to “reverse” and amplify muscle contractions in “active” stick insects (30, 83), similar tests have not been able to demonstrate reflex reversals in motor effects of the tibial campaniform sensilla.

In stick insects, these motor effects could contribute to cocontraction of the tibial flexor and extensor muscles in the stance phase of walking. Periods of coactivation of tibial muscles in walking are evident in myographic recordings of tibial muscles of the middle legs in stick insects (51) and cockroaches (49, 58). In addition, the period of cocontraction in cockroaches was demonstrated to depend on sensory feedback, as it was absent in denervated preparations that showed purely reciprocal motor bursting in tibial muscles (49). Coactivation of muscles is widely seen in walking and other behaviors in vertebrates, although the contribution of sensory feedback to cocontraction has not been extensively investigated (84–86). However, force feedback is now considered to be context dependent and to enhance or stabilize motor outputs at different joints depending on the anatomical location of the receptors and behavior of the animal (9, 87, 88). The present study suggests that sensory inputs that signal force may be adaptively used in natural behaviors of both invertebrates and vertebrates to similar advantage.

## GRANTS

This study was supported by West Virginia Clinical and Translation Science Institute Grant U54GM104942, Deutsche Forschungsgemeinschaft (German National Science Foundation) Grant BU 857-15, and National Science Foundation Grant 2015317.

## DISCLOSURES

No conflicts of interest, financial or otherwise, are declared by the authors.

## AUTHOR CONTRIBUTIONS

S.N.Z., C.J.D., and N.S.S. conceived and designed research; S.N.Z. and J.S. performed experiments; S.N.Z., C.J.D., and N.S.S. analyzed data; S.N.Z., C.J.D., N.S.S., A.B., and J.S. interpreted results of experiments; C.J.D. prepared figures; S.N.Z., C.J.D., and N.S.S. drafted manuscript; S.N.Z., C.J.D., N.S.S., A.B., and J.S. edited and revised manuscript; S.N.Z., C.J.D., N.S.S., A.B., and J.S. approved final version of manuscript.
